# Identification of *HsfB* Family in Peanut (*Arachis hypogea*) and Role of *AhHsfB1-5A* in High-Temperature Stress

**DOI:** 10.3390/plants15121768

**Published:** 2026-06-08

**Authors:** Qiuguo Shi, Wei Wang, Guangdong Zhao, Xiaoli Zhang, Wei Sun, Junming Gu, Siyu Chen, Guimin Li, Shucai Wang, Wenxuan Du, Mingjing Zhang, Xiaojun Hu

**Affiliations:** 1 College of Life Sciences, Linyi University, Linyi 276005, China; shiqiuguo@lyu.edu.cn (Q.S.); wangwei1226@lyu.edu.cn (W.W.); 15684278615@163.com (W.D.); 2 Linyi Institute of Industrial Technology for High-Quality Grain & Oil Crops and Deep Processing, Linyi Academy of Agricultural Sciences, Linyi 276003, China; 3 Laboratory of Plant Molecular Genetics & Crop Gene Editing, College of Life Sciences, Linyi University, Linyi 276005, China; 4 National Peanut Industrial Technology System Experimental Station, Linyi Academy of Agricultural Sciences, Linyi 276003, China

**Keywords:** abiotic stress, *Arachis hypogaea* L., bioinformatics analysis, *HsfB* subfamily, thermotolerance

## Abstract

Global warming-triggered heat stress severely restricts plant growth and crop productivity. Peanut (*Arachis hypogaea* L.), a vital oilseed and cash crop that is susceptible to high temperatures throughout its growth cycle, exhibits inhibited peg and pod development, growth retardation, and premature leaf senescence under heat stress, which ultimately causes substantial yield losses. Heat shock factors (Hsfs) serve as core regulatory modulators of plant abiotic stress tolerance, among which the HsfB subfamily exerts a critical function in thermotolerance modulation. Nevertheless, the biological functions of peanut *HsfB* genes remain largely uncharacterized. In the present study, a total of 16 *HsfB* subfamily members were identified from the peanut genome, possessing highly conserved gene structures and protein motifs. Phylogenetic analysis revealed that the peanut *AhHsfB* genes are classified into four distinct subfamilies. Chromosomal localization analysis indicated that these 16 *AhHsfB* genes are unevenly distributed across nine peanut chromosomes. Transcriptomic profiling demonstrated that the transcript levels of *AhHsfB* genes were significantly upregulated by 6- to 120-fold upon heat stress exposure. Subcellular localization and transcriptional activity assays further validated that AhHsfB1-5A is a nucleus-localized protein with intrinsic transcriptional activation activity. Ectopic overexpression of *AhHsfB1-5A* in *Arabidopsis thaliana* remarkably enhanced seed germination ability and antioxidant capacity under heat stress conditions, with a maximum 18.84% increase in green seedling rate. This study systematically characterizes the HsfB subfamily in peanut and elucidates the positive regulatory role of *AhHsfB1-5A* in plant thermotolerance. These findings deepen our understanding of the role of *HsfB* and provide valuable genetic resources for molecular breeding of heat-resistant peanut varieties.

## 1. Introduction

Global climate change has drastically increased the frequency and intensity of heat stress events, posing a severe threat to plant growth, development, and agricultural productivity worldwide [1]. To cope with abrupt temperature elevation, plants activate a conserved defensive mechanism termed the heat shock response (HSR) [2,3]. This response is mainly regulated by evolutionarily conserved transcription factors—heat shock factors (HSFs)—which can bind to heat shock elements (HSEs) in the promoters of heat shock proteins (HSPs), ROS scavenging system-related genes, proteases genes and other protective genes, thereby modulating the expression of these downstream genes to enhance plant thermotolerance [3,4,5].

Compared with animals, the heat shock factor (HSF) family in plants has undergone substantial expansion and is classified into three major subfamilies (HsfA, HsfB, and HsfC) based on the structural characteristics of their oligomerization domain (OD, also known as the HR-A/B region) [5,6]. Structurally, a typical plant HSF comprises a conserved N-terminal DNA-binding domain (DBD), an adjacent oligomerization domain (OD), nuclear localization/export signals (NLS/NES), and a C-terminal activator motif (AHA motif) [4,5,6]. The OD is located downstream of the DBD and contains two hydrophobic heptapeptide repeat regions, namely HR-A and HR-B [7]. Specifically, members of subfamily A and subfamily C harbor amino acid insertions between HR-A and HR-B, respectively, while no inserted sequence exists in these two regions in subfamily B members [8]. When plants are subjected to external high-temperature stress, HSFs in plants form homo- or heterotrimers via the coiled-coil structure formed by HR-A/B [9]. This oligomerization enables them to bind to heat shock elements (HSEs) in the promoters of heat shock proteins (HSPs), thereby inducing HSP expression [9]. Notably, the AHA motif is a unique signature specific to subfamily A HSFs and is absent from subfamilies B and C [10].

Subfamily A HSFs are well established as the master regulators of heat stress responses, capable of inducing the expression of stress-responsive genes [9], such as *SlHSFA2* and *SlHSFA7* in tomato (*Solanum lycopersicum*) [11,12]; *HSFA2*, *HSFA6a*, and *HSFA6b* in *Arabidopsis thaliana* [13,14]; *ZmHSF06* in maize (*Zea mays*) [15]; *BnaHsfA2* in rapeseed (*Brassica napus*) [16]; *TaHsfA2-10* in wheat (*Triticum aestivum*) [17]; and *CsHsfA1a* in citrus species [18]. In contrast, subfamily B HSFs are poorly understood, and are generally considered to function as co-regulators, acting as either transcriptional repressors or activators depending on their protein interactors [5,10]. For instance, *HsfB1* and *HsfB2*b in *Arabidopsis* act as repressors of heat-induced HSF expression, modulating acquired thermotolerance [19]. Interestingly, in tomato, *HsfB1* exhibits dual functionality, serving as both a transcriptional repressor and a co-activator [20]. However, the regulatory networks underlying the functions of HsfB remain poorly understood and warrant further investigation.

Peanut (*Arachis hypogaea* L.) is an economically important oilseed and cash crop worldwide, which accumulates rich reserves of vegetable oils, proteins, minerals, and vitamins. As peanut plants are susceptible to diverse abiotic stresses throughout their growth cycle, this crop serves as a precious genetic resource for the mining of stress-tolerance genes [21]. Heat stress occurring during the flowering and pod-setting stages significantly reduces flower number and pollen viability, impedes the translocation of dry matter to developing pods, and ultimately leads to seed shriveling and yield loss [22,23]. In addition, high-temperature stress aggravates *Aspergillus flavus* infection in peanut [24]. A total of 46 *AhHsf* genes have been identified in the peanut genome [25]. Of these genes, four members of the *AhHsfA* subfamily (*AhHsf5*, *AhHsf20*, *AhHsf24*, and *AhHsf30*) and two members of the *AhHsfB* subfamily (*AhHsf11* and *AhHsf35*) are markedly induced under drought and salt stress conditions. Heterologous overexpression of *AhHsf20* has been confirmed to significantly enhance salt tolerance in *Arabidopsis thaliana* [25]. A previous study revealed that 13 out of 17 peanut *AhHsf* genes are heat-inducible, among which two genes (*AhHsf2* and *AhHsf14*) specifically respond to *Aspergillus flavus* infection [24]. To date, the biological functions of *AhHsfB* family genes in peanut remain largely uncharacterized. In this study, we systematically identified the *AhHsfB* gene family in peanut; comprehensively analyzed its gene structures, promoter cis-elements, chromosomal distribution, phylogenetic relationships, and expression patterns; and functionally characterized *AhHsfB1-5A*.

## 2. Results

### 2.1. Phylogenetic Tree and Structure of Peanut AhHsf Subfamily B Genes

**Figure 1:**
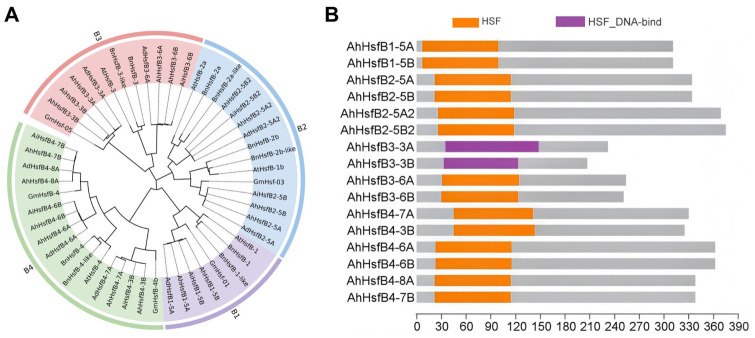
The phylogenetic tree and conserved domains of HsfB subfamily. (**A**) Phylogenetic tree of HSF subfamily B proteins from peanut and 5 other species. The fan-shaped regions of different colors represent the four subgroups B1, B2, B3, and B4. (**B**) Conserved domain analysis; purple represents the HSF_DNA-bind domain. The gray bars represent the full-length protein sequences, with the scale bar (0–390) indicating amino acid positions.

We identified 48 heat shock transcription factors in cultivated peanut from PeanutBase and NCBI through homology search (Figure S1), among which 16 belonged to subfamily B (Table S1). These genes are named according to their chromosomal locations and homologous sequences in other plant species. To elucidate the phylogenetic relationships and evolutionary characteristics of the AhHsf subfamily B proteins, a phylogenetic tree was constructed using the protein sequences of 52 HSF subfamily B proteins, including 16 AhHsfB members from cultivated peanut, 8 from its ancestral species *Arachis duranensis* (Ad), 8 from *Arachis ipaensis* (Ai), 5 from soybean (*Glycine max*, Gm), 5 from *Arabidopsis* (*Arabidopsis thaliana*, At), and 10 from rapeseed (*Brassica napus*, Bn). Based on the phylogenetic tree, the HSF subfamily B proteins could be clearly divided into four subgroups (B1, B2, B3, and B4) (Figure 1A). The four subgroups comprise two, four, four, and six cultivated peanut AhHsfB members, respectively.

To explore the sequence characteristics of the AhHsfB subfamily, conserved motifs, conserved domains, and gene structures were further investigated. A total of eight conserved motifs (Motif 1–8) were identified by analyzing 16 AhHsfB proteins using the MEME tool (Figure S2), among which Motif 1, Motif 2, and Motif 4 were the core motifs shared by all AhHsf subfamily B proteins. Combining the results of conserved domain analysis, Motif 1 and Motif 2 together constitute the HSF protein-specific DNA binding domain (HSF_DNA-bind) (Figure 1B), which serves as the molecular basis for HSF proteins to recognize and bind to the heat shock elements (HSEs) of downstream genes. The distribution of motifs exhibits distinct characteristics among the subgroups; for example, members of the B4 subgroup generally contain Motif 3, while members of the B2 subgroup mostly contain Motif 5 and Motif 6. Gene structure analysis shows that *AhHsfBs* contain only one intron (Figure S3), and the intron sequences of subgroup B2 are longer than those of the other subgroups. These results not only confirm the classification results of the phylogenetic tree but also indicate that the genes of subfamily B share a common evolutionary origin.

### 2.2. Chromosomal Localization and Duplication Analysis of Peanut AhHsfB Subfamily Genes

**Figure 2:**
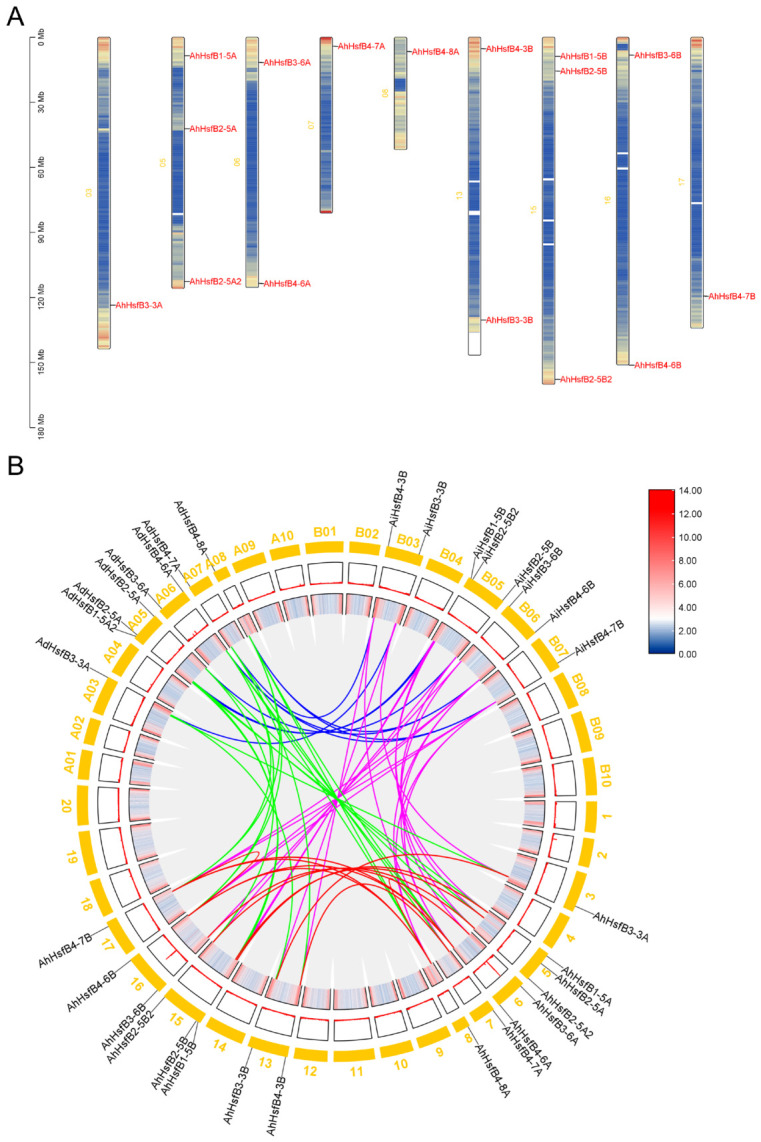
Chromosomal localization and collinearity analysis of *AhHsfB* subfamily genes in *Arachis hypogaea.* (**A**) Chromosomal localization of *AhHsfB* subfamily genes in *Arachis hypogaea*. The chromosomal regions with different colors indicate gene density, and the specific name and location of each gene are labeled. (**B**) Circos plot showing collinear relationships of HSFB subfamily genes between *Arachis hypogaea* (Ah) and its diploid progenitors (*Arachis duranensis, Ad*; *Arachis ipaensis, Ai*). Different colored lines represent collinear gene pairs between different species: red for *Arachis hypogaea* intraspecies, green for Ah vs. Ad, purple for Ah vs. Ai, and blue for Ad vs. Ai. The outer heatmap shows gene expression levels, with a color scale ranging from 2.00 to 14.00.

Chromosomal localization analysis showed that these 16 genes were not randomly distributed but were localized on nine different chromosomes (Chr. 3, 5, 6, 7, 8, 13, 15, 16, and 17), showing obvious chromosomal preference. Among them, chromosomes 5 and 15 were distribution hotspots, each containing three subfamily members; chromosomes 6, 13, and 16 each contained two genes, and the remaining chromosomes contained only one gene (Figure 2A). Collinearity analysis identified that a total of 12 pairs of collinear genes were detected within cultivated peanut, reflecting extensive gene retention and duplication between the A and B subgenomes of peanut after allopolyploidization (Figure 2B). There were a large number of collinear relationships between cultivated peanut and the diploid ancestral species, among which the number of collinear gene pairs between *Arachis hypogaea* and *Arachis ipaensis* (20 pairs) was higher than that between *Arachis hypogaea* and *Arachis duranensis* (18 pairs). In addition, nine pairs of collinear genes were also detected between the two ancestral species, indicating that the *HsfB* subfamily existed before the differentiation of diploid species. Most *AhHsfB* members retained one homologous copy each within *Arachis hypogaea*, *Arachis duranensis* and *Arachis ipaensis*, which is consistent with the typical retention pattern of allopolyploid plants after whole-genome duplication [26]. Among them, four members, *AhHsfB4-6A*, *AhHsfB4-6B*, *AhHsfB4-8*A, and *AhHsfB4-7B*, retained as many as seven collinear genes, while *AhHsfB3-6A*, *AhHsfB3-3B*, and *AhHsfB3-6B* retained only two collinear genes.

### 2.3. Expression Pattern of Peanut AhHsfB Subfamily Genes Under Extreme Temperatures and Drought Stress

**Figure 3:**
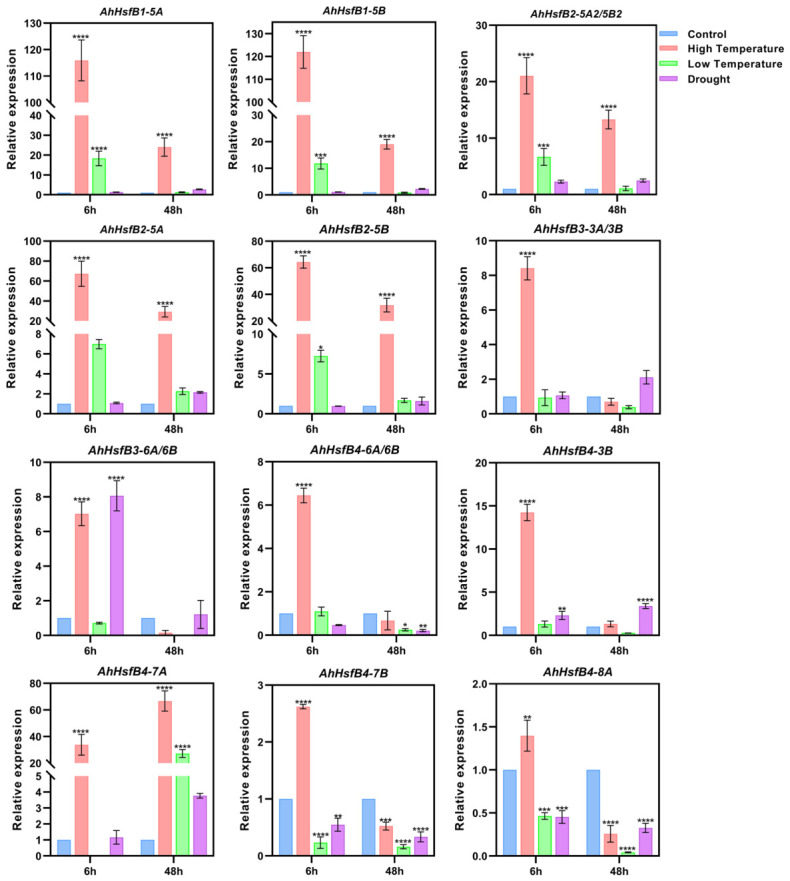
Expression pattern of peanut *AhHsfB* genes. qRT-PCR expression analysis of selected peanut *AhHsfB* genes under different abiotic stresses. Bar charts of different colors represent different stress treatments, respectively. The height of the bars indicates the relative expression level of genes (calculated by the 2^−∆∆Ct^ method), with 3 biological replicates set for each treatment. Note: * *p* < 0.05, ** *p* < 0.01, *** *p* < 0.001, **** *p* < 0.0001.

Heat stress response elements (STREs), low-temperature response elements (LTR) and drought response elements (MYB binding site, MBS) are commonly present in the promoter regions of peanut *AhHsfB* genes (Figure S4). Accordingly, we analyzed the expression profiles of these genes in response to high-temperature (42 °C), low-temperature (4 °C) and simulated drought stress (200 mM mannitol) (Figure 3). The results demonstrated that all *AhHsfB* genes exhibited a strong upregulation at 6 h after 42 °C treatment, and maintained high expression levels at 48 h except for *AhHsfB3-3B*, *AhHsfB3-6A, AhHsfB4-3B, AhHsfB4-6A/6B* and *AhHsfB4-8.* Besides heat stress, low-temperature treatment for 6 h markedly induced the expression of *AhHsfB1-5A*, *AhHsfB1-5B*, *AhHsfB2-5A*, *AhHsfB2-5A2* and *AhHsfB2-5B*. After 48 h of low-temperature exposure, the transcript level of *AhHsfB4-7A* was significantly elevated, whereas *AhHsfB4-6A/6B*, *AhHsfB4-7B* and *AhHsfB4-8* showed significant or highly significant downregulation. Only *AhHsfB4-3B* was positively responsive to 48 h of drought treatment. Interestingly, the expression levels of *AhHsfB4-6A/6B, AhHsfB4-7B* and *AhHsfB4-8A* were significantly repressed under both drought and low-temperature conditions.

### 2.4. AhHsfB1-5A Is a Nuclear-Localized Transcriptional Activator

**Figure 4:**
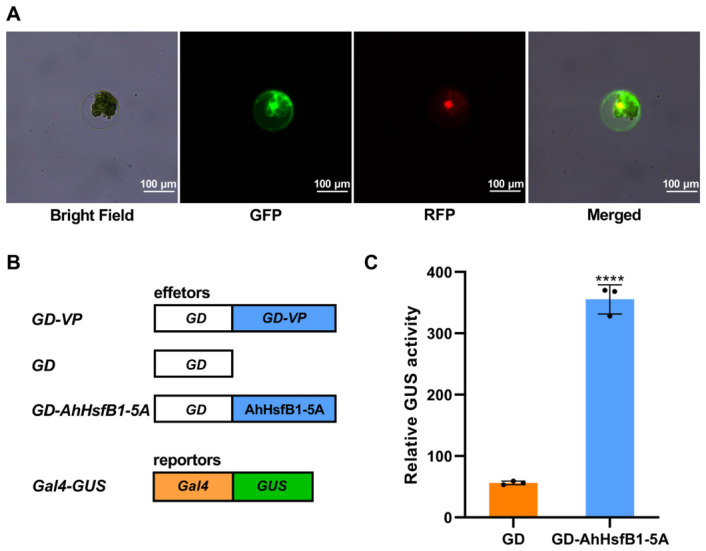
Subcellular localization and transcriptional activation activity analysis of AhHsfB1-5A. (**A**) Subcellular localization of AhHsfB1-5A. Observation under a laser confocal microscope showed that the GFP (green) and RFP (red) fluorescence signals completely overlapped (yellow), indicating that the AhHsfB1-5A protein is localized in the nucleus. (**B**) Schematic diagram of vector co-transfection for transcriptional activation activity detection. (**C**) Transcriptional activation activity of AhHsfB1-5A. Three biological replicates were set in the experiment. Note: **** *p* < 0.0001.

To understand the functions of HsfB subfamily members, we selected *AhHsfB1-5A* for further study. The *pUC35S::GFP-AhHsfB1-5A* fusion expression vector was constructed and co-transfected with the *NLS-RFP* nuclear indicator plasmid into isolated *Arabidopsis* protoplasts. After 22 h of incubation, the fluorescence signals were observed using a fluorescence microscope. The results showed that there was obvious overlap between GFP fluorescence and RFP fluorescence (Figure 4A), indicating that AhHsfB1-5A was predominantly localized in the nucleus.

Following the confirmation of AhHsfB1-5A localization in the nucleus, its transcriptional activity was further explored using the *Gal4:GUS* reporter gene, which can be activated by the *GD-VP* transcriptional activator. The *pUC35S::GD-AhHsfB1-5A* fusion expression vector was co-transfected with the *Gal4:GUS* reporter gene vector and *GD-VP* vector into *Arabidopsis* protoplasts (Figure 4B), with the co-transfection group of the *GD* empty vector, *Gal4:GUS* reporter gene vector and *GD-VP* vector as the blank control. The experimental results showed that in protoplasts co-transfected with *AhHsfB1-5A*, AhHsfB1-5A promoted the binding of *GD* to *Gal4-GUS*, thereby activating the expression of the GUS reporter gene and ultimately leading to a significant increase in GUS activity. Compared with the control *pUC35S::GD* group, the GUS activity of the *pUC35S::GD-AhHsfB1-5A* co-transfection group was significantly increased (Figure 4C), indicating that AhHsfB1-5A has transcriptional activation activity.

To determine the tissues in which *AhHsfB1-5A* functions, we analyzed the peanut multi-tissue transcriptome data from Professor Josh Clevenger’s team. The results showed that *AhHsfB1-5A* was highly expressed in all tissues except leaves, especially in the pericarp, fruit and root tissues (Figure S5). Meanwhile, we found that members of the B1 and B2 subgroups were highly expressed in all tissues, especially in reproductive organs. *AhHsfB3-6A/6B* exhibited significantly higher expression levels in nodules than in other tissues, while *AhHsfB4-7A/3B* was highly expressed in pods and seeds. The remaining six genes showed low expression levels across all examined tissues.

### 2.5. Overexpression of AhHsfB1-5A in Arabidopsis Thaliana Enhances Seed Germination Under Heat Stress

**Figure 5:**
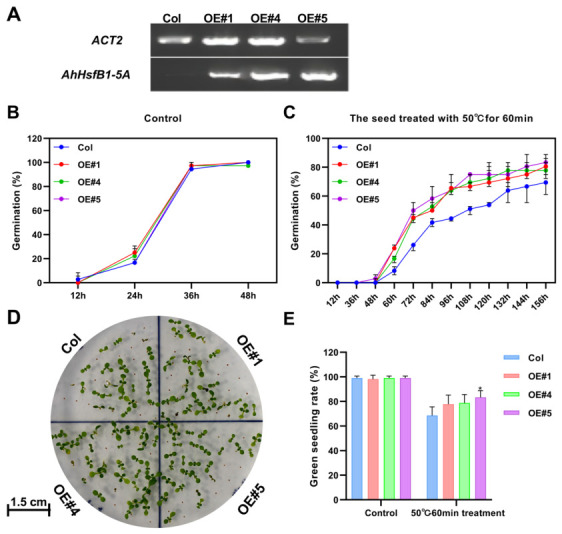
Effects of *AhHsfB1-5A* overexpression on *Arabidopsis* seed germination and tolerance to high-temperature stress. (**A**) Identification of *AhHsfB1-5A* overexpression in transgenic *Arabidopsis*. (**B**,**C**) Germination kinetic of seeds from wild-type *Col* and *AhHsfB1-5A* overexpression lines (OE#1, OE#4, OE#5) under normal conditions and 50 °C heat stress treatment. (**D**) Phenotype diagram of seeds from each line germinated on ^1^/_2_ MS medium for 156 h after heat stress treatment. (**E**) Statistics of the final germination rate of seeds from each line after heat stress treatment. Three biological replicates were set in the experiment. Note: * *p* < 0.05.

Wild-type *Arabidopsis thaliana* ecotype *Columbia* (*Col*) and T3-generation homozygous transgenic lines overexpressing *AhHsfB1-5A* (OE#1, OE#4, OE#5) were used to investigate seed germination (Figure 5A). Under normal growth conditions, nearly all seeds of both wild-type Col-0 and the *AhHsfB1-5A*-overexpressing lines germinated by 36 h after incubation, and the final germination rate of all lines remained at approximately 100%. This indicates that overexpression of *AhHsfB1-5A* has no effect on seed germination under normal conditions (Figure 5B). Following heat stress treatment at 50 °C, seed germination was inhibited in all lines, and the germination rate was markedly delayed, with germination rates stabilizing at 156 h of culture. Compared with wild-type *Col*, the inhibition of seed germination was significantly elevated in the *AhHsfB1-5A*-overexpressing lines, and their germination rates were significantly higher (Figure 5C). Statistical analysis revealed that the final germination rates of Col, OE#1, OE#4, and OE#5 after heat stress were 66.67%, 79.63%, 78.70%, and 83.33%, respectively (Figure 5E). These results demonstrate that overexpression of *AhHsfB1-5A* significantly improves the germination rate of *Arabidopsis* seeds under high-temperature stress and enhances seed thermotolerance (Figure 5D).

### 2.6. Overexpression of AhHsfB1-5A in Arabidopsis Thaliana Increases Survival Rate Under Heat Stress and Alleviates Oxidative Damage

**Figure 6:**
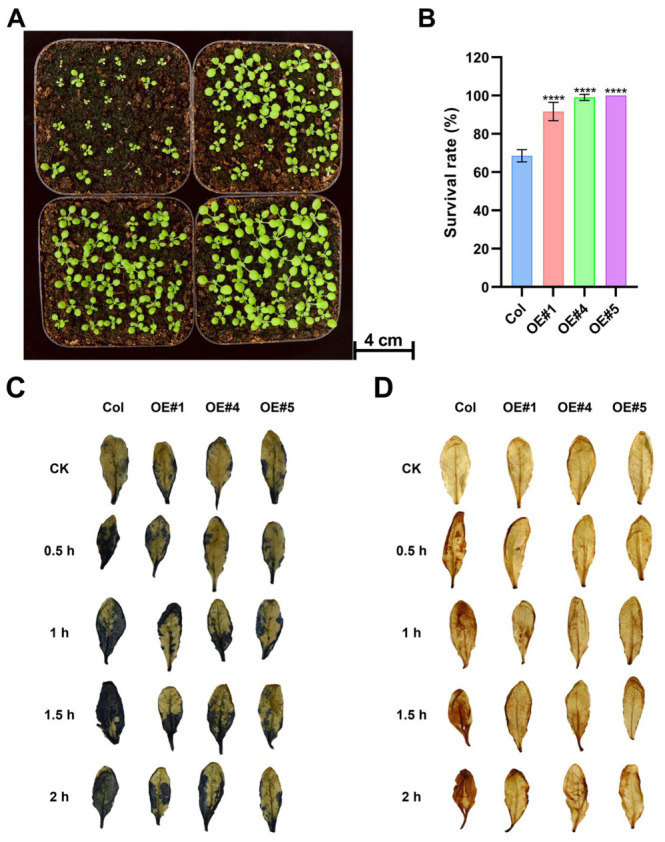
Effects of *AhHsfB1-5A* overexpression on the high-temperature stress tolerance of *Arabidopsis* seedlings. (**A**) Seedling phenotypes of wild-type *Col* and *AhHsfB1-5A* overexpression lines (OE#1, OE#4, OE#5) after 45 °C high-temperature stress treatment followed by 7 days of recovery growth. (**B**) Statistical analysis of seedling survival rates of each line after high-temperature stress treatment. Bar charts of different colors represent different lines, and the height of the bars reflects the difference in survival rate. Three biological replicates were set in the experiment. Note: **** *p* < 0.0001. (**C**) Detection of superoxide anion by NBT method. (**D**) Detection of peroxides by DAB method.

A seedling thermotolerance assay was performed to further characterize the heat tolerance conferred by *AhHsfB1-5A*. After heat stress treatment, the survival rate of wild-type seedlings declined significantly, whereas the survival rates of *AhHsfB1-5A*-overexpressing lines (OE#1, OE#4, and OE#5) did not decrease drastically and remained relatively high (Figure 6A). Statistical analysis showed that the survival rates of Col, OE#1, OE#4, and OE#5 lines following heat stress treatment were 68.52%, 91.67%, 99.07%, and 100%, respectively (Figure 6B). These results demonstrate that overexpression of *AhHsfB1-5A* significantly enhances the thermotolerance of *Arabidopsis* seedlings and strengthens plant resistance to heat stress.

To further assess the degree of damage to *Arabidopsis* seedlings under heat stress upon *AhHsfB1-5A* overexpression, 4-week-old wild-type *Col* and *AhHsfB1-5A* overexpression lines (OE#1, OE#4, OE#5) with consistent growth status were selected and subjected to gradient heat stress at 45 °C. ROS staining kits were used for staining. The results showed that with the extension of heat stress duration, the damage degree of all seedlings gradually increased, but the damage to wild-type plants was significantly more severe than that to *AhHsfB1-5A* overexpression lines (Figure 6C,D). These findings indicate that *AhHsfB1-5A* overexpression can significantly enhance the antioxidant capacity and reduce seedling damage in *Arabidopsis* and alleviate heat-induced oxidative damage to improve plant tolerance to heat stress.

## 3. Discussion

Heat shock transcription factors (HSFs) play crucial roles in response to heat stress as well as other abiotic stresses. Compared with animals (which possess only 1-3 HSFs) and fungi, plants harbor a remarkably expanded HSF family—for instance, 21 in *Arabidopsis thaliana* [4], 25 in rice (*Oryza sativa*) [22,27], and 26 in tomato (*Solanum lycopersicum*) [28]. A total of 4208 HSF genes identified from the genomes of 166 plant species were systematically analyzed. The results revealed that HSF genes originated in the early evolutionary stage of green algae, and genome-wide genetic variations occurred during plant evolution [29]. This phenomenon reflects the adaptive evolution of plants to cope with diverse environmental stresses [6]. In this study, we identified 48 *HSF* genes in cultivated peanut (*Arachis hypogaea*), two more than the number previously reported by Wang et al. (2023), and 31 more than reported by Wang et al. (2017) [24,25]. These two additional members belong to the HsfA subfamily, designated *AhHsfA5-3A* (which positively responds to high-temperature stress) and *AhHsfA1-6B*. With the continuous improvement of the peanut genome assembly, it is likely that more *HSF* genes with important biological functions will be identified in the future.

Members of the HsfB subfamily have traditionally been classified as transcriptional repressors [6,19,30]. However, recent studies have revealed considerable functional diversity and complexity within the HsfB subfamily, with some members even exhibiting transcriptional activation activity [20,31,32]. In this study, compared with the control *pUC35S::GD* group, the GUS activity in cells co-transfected with *pUC35S::GD-AhHsfB1-5A* and the *Gal4:GUS* reporter gene vector was significantly increased (Figure 4C), indicating that AhHsfB1-5A possesses transcriptional activation activity. Thus, AhHsfB1-5A was verified to function as a transcriptional activator, which supports these recent research advances. Notably, we identified the LFGV motif in the C-terminal region of all 16 AhHsfB members in peanut. Several studies have proposed that under stress conditions, the C-terminal region of HsfB members (e.g., *HsfB1*) undergoes structural folding, which shields the conserved LFGV repression motif and exposes key interaction interfaces to recruit co-activators, thereby conferring transcriptional activation capacity [6,20,33]. Other studies have demonstrated that HsfBs can form distinct conformational structures upon binding to diverse HSE cis-elements, enabling them to repress specific target genes while activating others [4,6,20]. Nevertheless, the specific molecular mechanism underlying the transcriptional activation regulation of AhHsfB1-5A remains to be further elucidated.

*AhHsfB1-5A* is mainly expressed in the pericarp, fruit, and root tissues. In the report by Wang et al. (2017), *AhHsfB1-5A* (designated as *AhHsf9*) was also highly expressed in flower tissues [24]. Quantitative real-time PCR (qRT-PCR) analysis showed that the expression level of *AhHsfB1-5A* was extremely significantly increased after treatment at 42 °C for 6 h and 48 h, which is consistent with the report by Wang et al. (2017) [24]. However, it showed no obvious response to drought treatment, which is consistent with the results reported by Wang et al. (2023) (where it was designated as *AhHsf10*) [25]. Therefore, in the present study, we only investigated the heat resistance of *AhHsfB1-5A*-overexpressing transgenic *Arabidopsis thaliana*. The germination rate of *Arabidopsis* seeds overexpressing *AhHsfB1-5A* was significantly higher than that of the control under 50 °C treatment, with less oxidative damage, indicating that *AhHsfB1-5A* can enhance the heat resistance of plants. In potato, *StHsfB5* is induced by heat stress, translocates into the nucleus, binds to the promoters of *sHSP17.6*, *sHSP21*, and *HSP80*, directly activates their expression, and the overexpression lines show significantly improved heat resistance, thus being defined as a transcriptional “co-activator” [32,33]. In rice, *OsHsfB2c* is induced by heat stress and H_2_O_2_, binds to the promoters of *HSP70* and *HSP90*, and enhances rice heat resistance [22]. We have not conducted in-depth research on the regulatory mechanism of peanut HSFs so far, and new insights will surely be gained through further in-depth studies in the future.

In addition to participating in heat stress response, *HsfB* is also involved in other stress responses and developmental regulation. *Arabidopsis thaliana* HsfB1 has a “dual function”: it inhibits *HSP101* expression under normal temperature to restrict growth; under drought conditions, it interacts with the translation factor eIF3g1, converts into an activator, up-regulates *HSP101*, enhances drought resistance, and balances growth and stress tolerance [34,35]. *Arabidopsis HsfB2b* is involved in the regulation of ion homeostasis under salt stress, and its mutant is sensitive to salt [35]. *Arabidopsis HsfB1* and *HsfB2b* regulate seed maturation and dormancy; their overexpression delays seed germination, while the mutant seeds show enhanced vitality [19,35]. Wheat *TaHsfC3B* is induced by drought and ABA; its overexpression improves the water retention capacity and antioxidant enzyme activity of transgenic *Arabidopsis*, and reduces ROS accumulation [36]. Research on the functions of HSFs in peanut is far from adequate. Wang et al. (2023) reported that *AhHsf20* (a member of the A2 subfamily) can enhance salt tolerance [25], and this study is the first to report the functions of the peanut *AhHsfB* gene family.

This study performed a systematic analysis of the HsfB subfamily in peanut, and a total of 16 *AhHsfB* members were identified. These members, together with their homologous genes from *Arachis duranensis*, *Arachis ipaensis*, *Arabidopsis thaliana*, *Glycine max* and *Brassica napus*, were divided into four subgroups. We further performed comprehensive bioinformatics analysis and expression pattern analysis on peanut HsfB subfamily genes, and verified the biological function of *AhHsfB1-5A*. The results indicated that *AhHsfB* genes may exert essential regulatory effects on abiotic stress responses and developmental processes in peanut. Notably, the expression of *AhHsfB1-5A* was strongly induced by high-temperature stress, and this gene could significantly enhance the thermotolerance of transgenic *Arabidopsis thaliana*. In conclusion, the present findings lay a foundation for further exploring the precise biological functions of *AhHsfB* genes and provide novel genetic resources for stress resistance breeding in peanut.

## 4. Materials and Methods

### 4.1. Experimental Materials and Data Download

Peanut cultivar Haihua 1 (*Arachis hypogaea* L.) was grown in a light incubator under a 16 h light/8 h dark photoperiod at 22 ± 2 °C with 70 ± 5% relative humidity and routine watering. Fresh peanut leaves were sampled, frozen rapidly in liquid nitrogen, and stored at −80 °C. Total RNA was extracted using the FastPure Universal Plant Total RNA Isolation Kit (Vazyme Biotech, Nanjing, China) and reverse-transcribed into cDNA with the HiScript III RT SuperMix for qPCR (+gDNA wiper) (Vazyme Biotech, Nanjing, China). Wild-type *Arabidopsis thaliana* ecotype Col (Col-0) and *AhHsfB1-5A* overexpression lines were cultivated under identical conditions for transgenic identification, subcellular localization assay, transcriptional activity analysis, and thermotolerance evaluation [37].

Genomic data of peanut diploid ancestors *Arachis duranensis* (Ad, PI 475845) and *A. ipaensis* (Ai, PI 468323), as well as HSF sequences of *Glycine max* and *Brassica napus*, were downloaded from NCBI (https://www.ncbi.nlm.nih.gov/, accessed on 4 June 2026). *Arabidopsis* HSF sequences were obtained from TAIR (https://www.arabidopsis.org/, accessed on 4 June 2026). Cultivated peanut genomic and multi-tissue transcriptome data were retrieved from PeanutBase (https://peanutbase.org/, accessed on 4 June 2026).

Vectors used included *pUC19* (target fragment cloning/sequencing), *pZP211* (overexpression vector construction), *pUC35S::GFP* (subcellular localization fusion vector), and *pUC35S::GD* (transcriptional activity fusion vector). *Gal4-GUS* reporter vector, *GD-VP* transcriptional activator vector, and *NLS-RFP* nuclear indicator plasmid were preserved in the laboratory.

### 4.2. Identification and Physicochemical Property Analysis of Peanut AhHsf Subfamily B Genes

Candidate *AhHsf* genes were retrieved from PeanutBase. Conserved domains were analyzed using SMART [38] and NCBI CDD to confirm valid *AhHsf* genes with a DNA Binding Domain (DBD) [39]. AhHsfB subfamily members were screened by the insertion sequence between HR-A/HR-B domains and phylogenetic tree analysis, and named according to subgroup and chromosomal localization.

Physicochemical properties (amino acid number, molecular weight, isoelectric point, instability index, aliphatic index) of 16 AhHsfB subfamily proteins were analyzed via TBtools Protein Parameter Calc (v2.458) [39]. Subcellular localization was predicted using CELLO v2.5 [40].

### 4.3. Phylogenetic, Conserved Motif and Gene Structure Analysis

HSF B subfamily protein sequences from peanut; its diploid ancestors (Arachis *duranensis*, Arachis *ipaensis*); and *Glycine max*, *Arabidopsis thaliana*, and *Brassica napus* were aligned with ClustalX [41]. The phylogenetic tree was constructed using MEGA 12 based on the Maximum Likelihood (ML) method with 1000 bootstrap replicates [42].

Conserved motifs of AhHsfB subfamily proteins were analyzed via MEME (max 8 motifs, 6–50 amino acids) [43]. Conserved domains were visualized by TBtools (v2.458). Gene structure diagrams were drawn with TBtools (v2.458) [40] using genomic and CDS sequences from PeanutBase.

### 4.4. Chromosomal Localization and Collinearity Analysis

Chromosomal localization of AhHsfB subfamily members was obtained from PeanutBase, and the localization map was drawn by TBtools (v2.458). Collinearity analysis of *AhHsfB* subfamily genes between cultivated peanut (*Arachis hypogaea*) and its diploid ancestors (*Arachis duranensis*, *Arachis ipaensis*) was performed with MCScanX, and the circular collinearity map was visualized by TBtools [40].

### 4.5. Promoter Cis-Acting Element Analysis

Promoter regions of 2000 bp in length upstream of the ATG initiation codon of *AhHsfB* subfamily genes were extracted from the database. Cis-acting elements were predicted via PlantCARE (https://bioinformatics.psb.ugent.be/webtools/plantcare/html/, accessed on 4 June 2026) [44], focusing on abiotic stress-related elements (STRE, LTR, MBS, MYB, ABRE). A bar chart of stress-responsive element counts was drawn by GraphPad Prism 9 [45].

### 4.6. Gene Expression Pattern Analysis

Based on peanut full-life-cycle multi-tissue transcriptome data, AhHsfB subfamily expression levels were extracted, standardized, and clustered by TBtools (v2.458) to generate a spatial expression heatmap.

Representative members were selected for qRT-PCR verification. Total RNA was isolated from different tissues and leaves treated with 42 °C, 4 °C or 200 mM mannitol for 6 h and 48 h using the FastPure Universal Plant Total RNA Isolation Kit (Vazyme Biotech, Nanjing, China), and the obtained RNA was subsequently reverse-transcribed into cDNA with the HiScript III RT SuperMix for qPCR (+gDNA wiper) (Vazyme Biotech, Nanjing, China). Specific primers were designed (Table S2) with peanut *Actin* as the internal reference. qRT-PCR was performed with the SYBR^®^ Premix Ex Taq™ II kit (Takara Bio Inc, Beijing, China) The program was 95 °C for 30 s; 40 cycles of 95 °C for 5 s and 60 °C for 30 s, followed by melting curve analysis. Relative expression was calculated by the 2^−∆∆Ct^ method, and bar charts were drawn by GraphPad Prism 9 [45] (3 biological/technical replicates).

### 4.7. Construction of Overexpression Vector and Acquisition of Transgenic Arabidopsis

Specific primers with restriction enzyme sites were designed for the *AhHsfB1-5A* CDS. The target fragment was amplified from peanut cDNA with a high-fidelity PCR enzyme (Vazyme Biotech, Nanjing, China), cloned into *pUC19::HA*, and transformed into *E. coli* DH5α (Weidi Biotechnology, Shanghai, China). Positive clones were verified by sequencing. The target fragment and *pZP211* vector were double-digested, recovered, and ligated to construct the *35S::AhHsfB1-5A* overexpression vector, which was verified by digestion and sequencing.

The overexpression vector was transformed into *Arabidopsis* Col via the floral dip method. T0 seeds were screened on ^1^/_2_ MS medium (50 μg/mL kanamycin, 100 μg/mL carbenicillin). T1 lines with a 3:1 green/yellow seedling ratio were selected, and T2 seeds were screened to obtain T3 homozygous lines. Target gene integration and expression were verified by PCR using leaf cDNA.

### 4.8. Subcellular Localization and Transcriptional Activity Analysis

The target fragment without a stop codon was cloned into *pUC35S::GFP* to construct the *pUC35S::GFP-AhHsfB1-5A* vector (verified by digestion/sequencing). *Arabidopsis* protoplasts were isolated and co-transfected with the vector and *NLS-RFP* [46]. After 22 h of dark culture at 22 °C, GFP/RFP distribution was observed by a laser confocal microscope [47,48]. The *pUC35S::GD-AhHsfB1-5A* effector vector *(pUC35S::GD* as control), *Gal4:GUS* reporter vector, and *GD-VP* activator vector were co-transfected into protoplasts. After 17 h of dark culture at 22 °C, GUS activity was detected by a microplate reader [49,50].

### 4.9. High-Temperature Tolerance Verification

Wild-type and T3 homozygous overexpression (OE#1, OE#4, OE#5) *Arabidopsis* seeds were disinfected. The heat treatment group was incubated at 50 °C for 60 min, and then both groups were spotted on ^1^/_2_ MS medium. The germination rate was counted every 12 h. Five-day-old seedlings with consistent growth were divided into control and high-temperature groups (45 °C for 90 min). After 7 days of recovery growth, seedling phenotype and survival rate were recorded [51]. Four-week-old wild-type Col *Arabidopsis thaliana* and T3 homozygous *AhHsfB1-5A* overexpression lines were subjected to heat treatment at 45 °C for 0.5 h, 1 h, 1.5 h, and 2 h, respectively. Leaves were then excised and stained using ROS staining kits (NBT and DAB methods, Solarbio Science & Technology Co., Ltd., Beijing, China) to observe the staining intensity, which reflects the degree of leaf damage.

### 4.10. Data Statistics

All experiments had 3 biological replicates. Data were expressed as Mean ± SD. Difference significance analysis and graphing were performed by GraphPad Prism 9 (* *p* < 0.05, ** *p* < 0.01, *** *p* < 0.001, **** *p* < 0.0001).

## Data Availability

The original contributions presented in this study are included in the article/Supplementary Material. Further inquiries can be directed to the corresponding authors.

## Supplementary Materials

The following supporting information can be downloaded at https://www.mdpi.com/article/10.3390/plants15121768/s1: Table S1: Analysis of physicochemical properties and subcellular localization prediction of the AhHSF B subfamily proteins in peanut (*Arachis hypogaea*). Table S2: The marker gene primers of fluorescence qPCR. Table S3: The table of motif base sequences. Figure S1: Conserved DNA-binding domains and HR-A/HR-B domains of the AhHsf in peanut (*Arachis hypogaea*). Figure S2: Conserved motif analysis of Hsf subfamily B. Figure S3: Gene structures of Hsf subfamily B. Figure S4: Number of abiotic stress-responsive elements in the promoter regions of AhHsf B family genes in *Arachis hypogaea*. Figure S5: Heatmap of expression levels of HSF subfamily B genes in peanut (*Arachis hypogaea*) across different tissues.
