# Supplementary material for: Identification of HsfB Family in Peanut (Arachis hypogea) and Role of AhHsfB1-5A in High-Temperature Stress
Source: Plants (Basel). 2026 Jun 8;15(12):1768. doi: 10.3390/plants15121768 (PMC13307298; doi:10.3390/plants15121768)
Supplement: Supplementary file 1 [file plants-15-01768-s001.zip › Supplementary Figure S2.pdf]

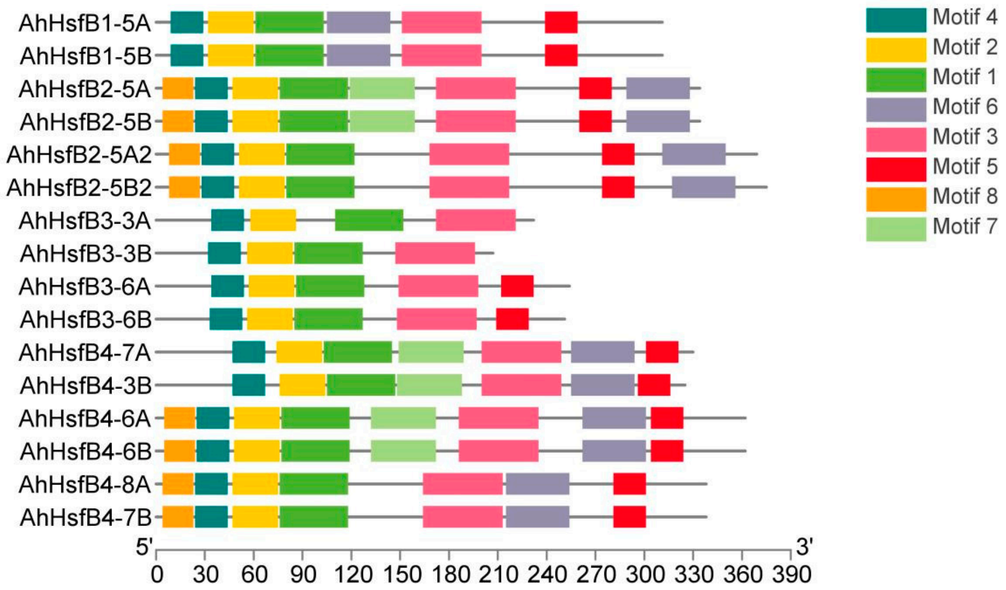

Supplementary Figure S2: Analysis of conserved motifs in HSF subfamily B.
Squares with distinct colors correspond to Motifs 1 – 8.
