# Supplementary material for: Identification of HsfB Family in Peanut (Arachis hypogea) and Role of AhHsfB1-5A in High-Temperature Stress
Source: Plants (Basel). 2026 Jun 8;15(12):1768. doi: 10.3390/plants15121768 (PMC13307298; doi:10.3390/plants15121768)
Supplement: Supplementary file 1 [file plants-15-01768-s001.zip › Supplementary Figure S4.pdf]

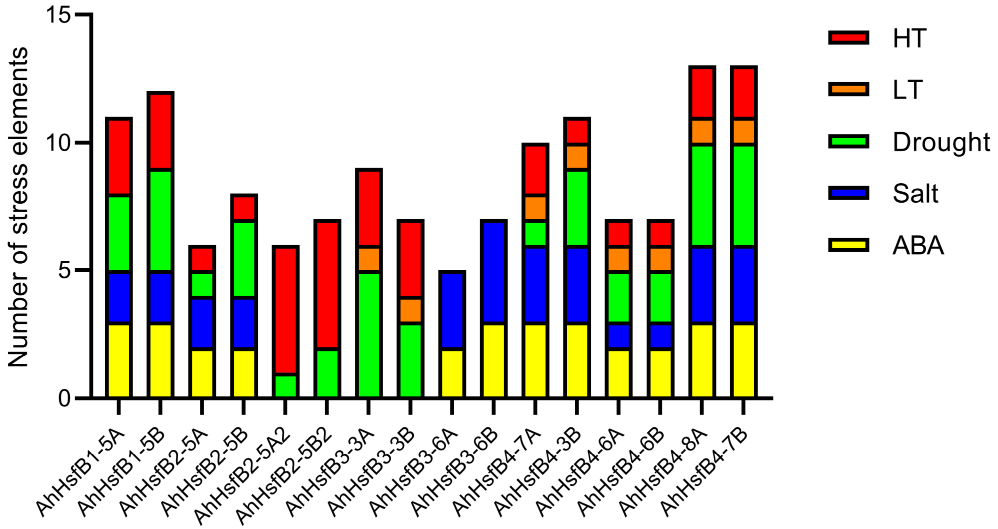

Supplementary Figure S4: Statistics of abiotic stress-responsive cis-elements in the promoter regions of peanut AhHSF-B family genes

The bar chart displays five types of stress-related cis-elements in the 2000 bp upstream sequences of ATG among 16 AhHSF-B members predicted by PlantCARE. Colored bars represent different elements: red (STRE, heat stress), green (LTR, low temperature), blue (MBS, drought), purple (MYB, salt stress), and yellow (ABRE, abscisic acid).
