# Supplementary material for: Identification of HsfB Family in Peanut (Arachis hypogea) and Role of AhHsfB1-5A in High-Temperature Stress
Source: Plants (Basel). 2026 Jun 8;15(12):1768. doi: 10.3390/plants15121768 (PMC13307298; doi:10.3390/plants15121768)
Supplement: Supplementary file 1 [file plants-15-01768-s001.zip › Supplementary Figure S5.pdf]

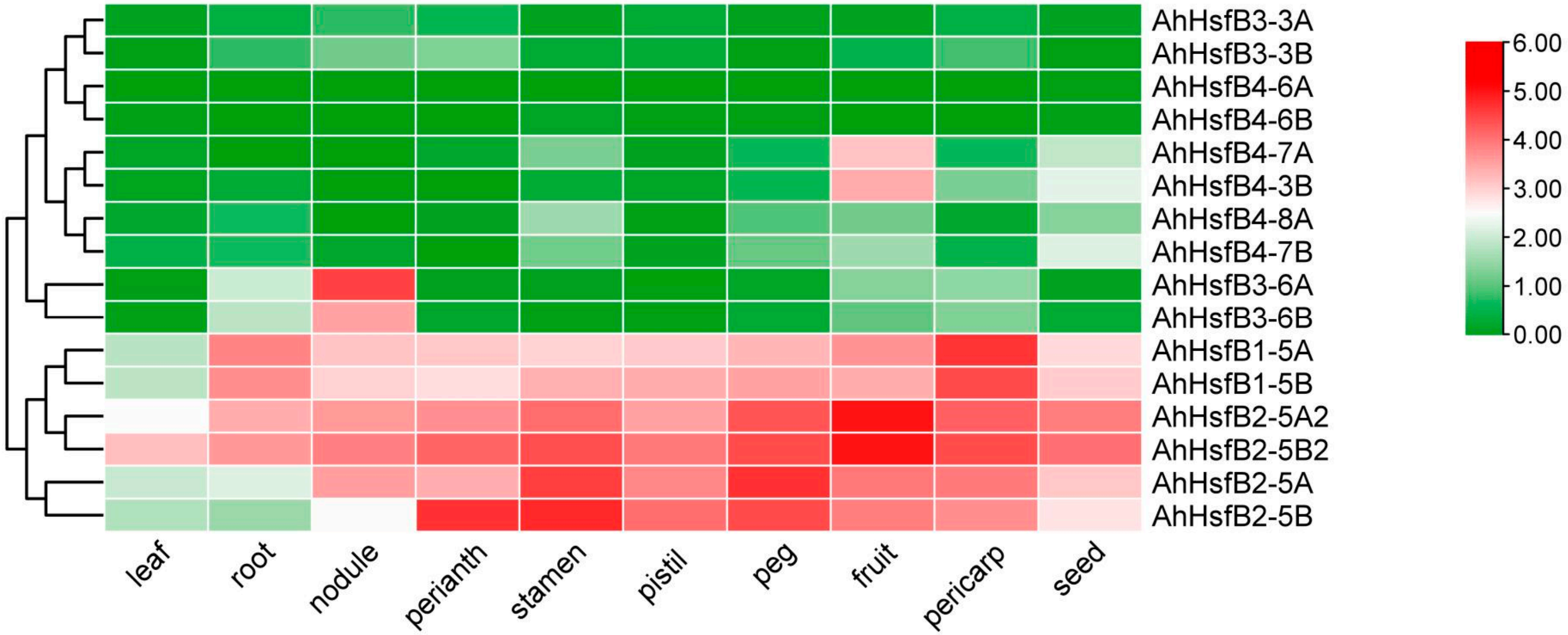

Supplementary Figure S5: Heatmap of expression levels of HSF subfamily B genes in peanut (*Arachis hypogaea*) across different tissues
