# Supplementary material for: Identification of HsfB Family in Peanut (Arachis hypogea) and Role of AhHsfB1-5A in High-Temperature Stress
Source: Plants (Basel). 2026 Jun 8;15(12):1768. doi: 10.3390/plants15121768 (PMC13307298; doi:10.3390/plants15121768)
Supplement: Supplementary file 1 [file plants-15-01768-s001.zip › Supplementray Table S2.pdf]

Supplementary Table S2 Primer sequences for quantitative real-time PCR (qRT-PCR)

Primer name	sequence 5'-3'
AhHsfB1-5A-F	CACAAGAGAGAGCGAGAAGGCC
AhHsfB1-5A-R	TGTCACGCCTTGTCTTCAACCT
AhHsfB1-5B-F	ACAACAACCACAAGAGAGGGCG
AhHsfB1-5B-R	ATGTCACGCCTCCTCTTCAACC
AhHsfB2-5A-F	CACAATCAAACGCAAACACAAAGGA
AhHsfB2-5A-R	AAACACCGCCTTTCCCAATTCG
AhHsfB2-5B-F	AATCAAACGCAAACACAAAGGCAA
AhHsfB2-5B-R	AAACACCGCCTTTCCCAATTCG
AhHsfB2-5A2/5B2-F	AGATTGCCAGAACCATCGCGAT
AhHsfB2-5A2/5B2-R	GCACGGACTCTGATTCTCTGCA
AhHsfB3-6A/6B-F	GCAGGTGAAGAGGAAGAGAGACG
AhHsfB3-6A/6B-R	ACCACATTAATAGCACAAGAGGGAGG
AhHsfB4-6A/6B-F	TGCAAGACGAAGCTATTTGGGGT
AhHsfB4-6A/6B-R	AGGAGGCATGAGATTCAATCCCA
AhHsfB4-7A-F	CAAAGCTTGTGGAGTTGGATTATTCATC
AhHsfB4-7A-R	AGGAACACCAAAGAGCTTCACTGA
AhHsfB4-7B-F	GGATGACCTTGGCTTGAATCTCATG
AhHsfB4-7B-R	TCACTCATCTCACGCACACACA
AhHsfB4-8A-F	GCGCGTTTGATTTTGGAGAAGGA
AhHsfB4-8A-R	CACACACACGTACGTTTCCATCA
AhHsfB3-3A/3B-F	ACCACCAAAATAAAAGTCGGGGAC
AhHsfB3-3A/3B-R	AAAGCCGTTGTTGCATGTTGGT
AhHsfB4-3B-F	ACCTGCTTCTTCTCCCTCTCATC
AhHsfB4-3B-R	AGGAACACCAAAGAGCTTCACTGA
Ahactin-F	GATTGGAATGGAAGCTGCTG
Ahactin-R	CGGTCAGCAATACCAGGGAA
