# Supplementary material for: Identification of HsfB Family in Peanut (Arachis hypogea) and Role of AhHsfB1-5A in High-Temperature Stress
Source: Plants (Basel). 2026 Jun 8;15(12):1768. doi: 10.3390/plants15121768 (PMC13307298; doi:10.3390/plants15121768)
Supplement: Supplementary file 1 [file plants-15-01768-s001.zip › Supplementray Table S3.pdf]

Supplementary Table S3 The table of motif base sequence

| Motif name | Sequence |
| --- | --- |
| Motif 1 | 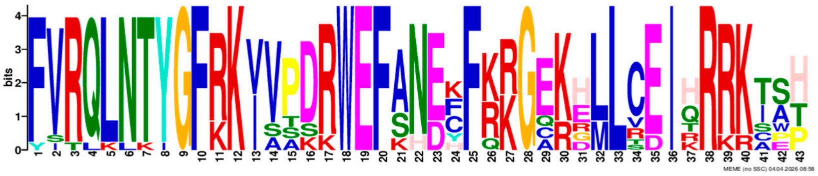 |
| Motif 2 | 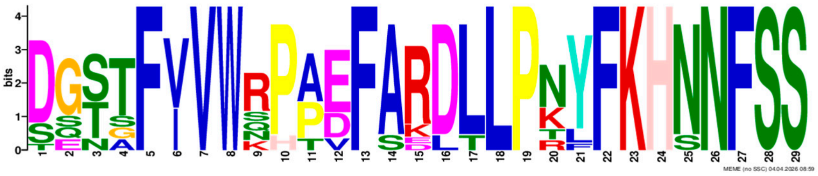 |
| Motif 3 | 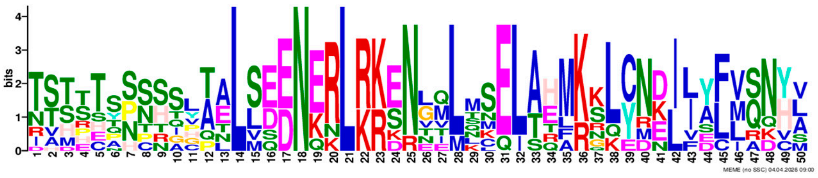 |
| Motif 4 | 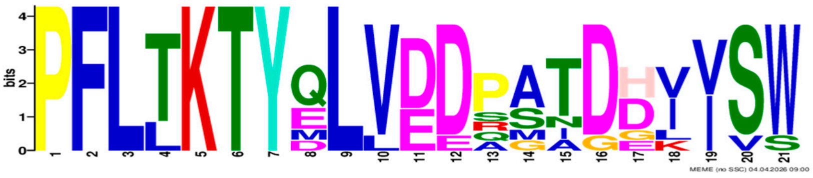 |
| Motif 5 | 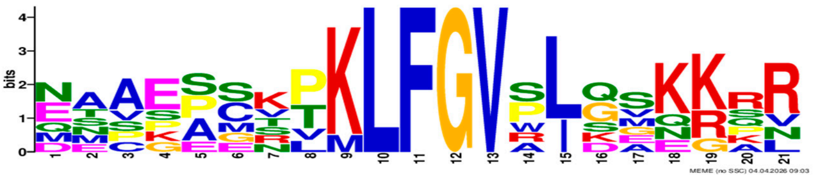 |
| Motif 6 | 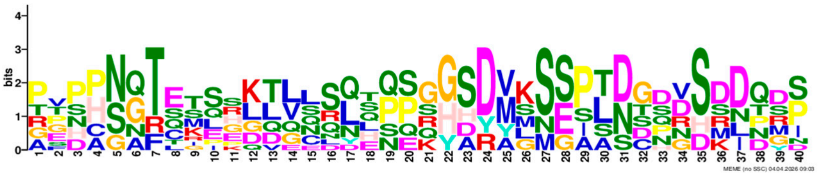 |
| Motif 7 | 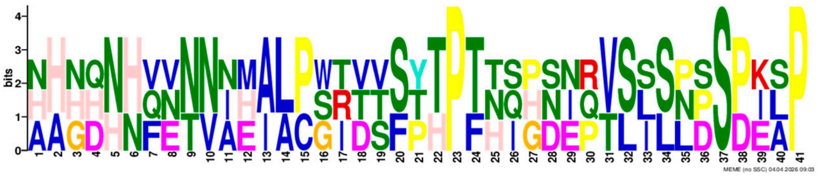 |
| Motif 8 | 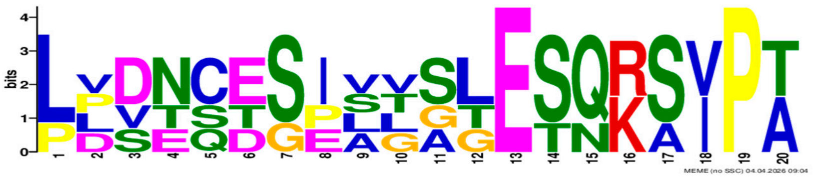 |
